# Dr. Carrington MacFarlane

**Published:** 1914-11

**Authors:** 


Dr. Carrington MacFarlane, P. & S. 1861, died in Oswego, August 12, aged 77. He was Asst. Surg. of the 81st and 115th N. Y. Volunteers, during the Civil War. He was one of the organizers of the City Hospital and for a long time a member of the staff.

